# The problem with confidence: too much and too little results in poorer achievement, inner conflict, and social inhibition

**DOI:** 10.3389/fpsyg.2023.960013

**Published:** 2023-05-18

**Authors:** Heather E. Douglas, Mitchell L. Cunningham, Jessika Tisdell, Justin Arneson

**Affiliations:** ^1^School of Psychological Sciences, The University of Newcastle, Newcastle, NSW, Australia; ^2^School of Psychology, The University of Sydney, Sydney, NSW, Australia; ^3^General Mills, Minneapolis, MN, United States

**Keywords:** ability, confidence, personality, response surface analysis, employed adults

## Abstract

**Introduction:**

Confidence is defined as the feelings and thoughts people have during a task that result in judgments about their performance. Evidence suggests that confidence is trait-like, but thus far research on the relative match between confidence and accuracy has been primarily restricted to over-confidence effects, and subject to the methodological flaws involved with using difference scores. We sought to answer an exploratory question in this research, whether discrepancies in ability and confidence in either direction reliably predicted individual differences on a broad-spectrum and commercially available personality test, the California Psychological Inventory (CPI260).

**Methods:**

Participants were 220 employed adults who had previously taken the CPI260 for career development purposes. They were invited to complete a measure of cognitive ability and confidence in return for feedback on the same. Data were modeled using polynomial regression and response surface analysis, to determine whether and how CPI260 personality traits were associated with matches or mismatches between accuracy and confidence in the same test.

**Results:**

We identified negative curvilinear effects along the line of disagreement for four CPI260 scales, suggesting that both under- and over-confidence were associated with personality.

**Discussion:**

In contrast to our expectations, individuals who were under-confident and those who were over-confident had lower achievement potential, less social confidence, and more inner conflict than other individuals in this sample. Although preliminary, these findings suggest that both over-confident and under-confident individuals are aware of potential weaknesses that impede their functioning.

## 1. Introduction

Confidence is a metacognitive experience, defined as the feelings and subsequent judgments people make during task performance (Efklides, 2006). Confidence is typically measured by embedding questions into ability tests, asking participants to rate their confidence that they answered the preceding question correctly. Such a method allows calculation of accuracy, average confidence across items, and discrepancies between the two indices to be examined. Discrepancies between accuracy and confidence judgments on the same task reflecting over-confidence are a robust finding in the extant literature (Pallier et al., 2002; Humberg et al., 2019), with these discrepancies widely thought to influence effective decision-making. The over-confident might attempt tasks that are beyond their capabilities, refuse the available assistance, and fail to detect the signs that decisions are unwarranted (Parker and Fischhoff, 2005). For example, Jackson et al. (2017) demonstrated that individuals with higher confidence tended to be more decisive. Jackson et al. (2017) found that the more decisive individuals initiated more target actions, but rather than making more accurate decisions, they were instead more reckless. These reckless decisions led to lost marks on cognitive tests, more damage accrued in an experimental firefighting decision making task, and more patient deaths in a medical decision-making scenario. Campbell et al. (2004) further found that overconfidence was associated with poorer performance on a betting task indicative of risk taking. In contrast, those exhibiting under-confidence might doubt their decision-making capability, as evidenced by increased deferment to others and hesitation on decisions where they are clearly correct (Parker and Fischhoff, 2005). Jackson and Kleitman (2014) used a fictitious medical scenario to demonstrate that under-confident individuals tended toward hesitation in situations where confident decisions and subsequent action were critical to the successful treatment of a fictitious patient (Jackson and Kleitman, 2014). Jackson et al. (2016) found that those who were less confident in their abilities more frequently avoided incorrect decisions in heuristics and biases tasks and averted riskier gambles in a betting task. These results have implications for organizations (Meikle et al., 2016), such that under conditions where the use of cognitive shortcuts is problematic, we might prefer a more cautious, or under-confident, decision maker. In contrast, under conditions where quick decisions using cognitive shortcuts are more beneficial or where the costs of hesitation are greater than taking risks, we might consider an individual who is measurably over-confident.

Evidence has accumulated for the role of individual differences in producing confidence bias, such that individuals reliably differ from one another in how much confidence bias they express (Pallier et al., 2002; Acker and Duck, 2008; Jackson et al., 2016, 2017; Cunningham et al., 2018). Given the evidence indicating stable individual differences in confidence bias, we might expect this individual level variation to be associated with other stable person characteristics. Linking confidence biases with prominent models of personality would give us some insight into the characteristics of individuals who are under- vs. over-confident. These insights are important because personality has been described as an individual’s default behavioral settings (Rebele et al., 2021). Understanding the default behaviors of those who are under- and over-confident alike might allow us to intervene in the problematic decision-making behaviors that confidence biases produce. Confidence itself is associated with key individual difference variables reflecting metacognitive knowledge, personality, and achievement (Pallier et al., 2002; Campbell et al., 2004; Kleitman and Stankov, 2007; Jain and Bearden, 2011; Buratti et al., 2013; Kasperski and Katzir, 2013; Burns et al., 2016). However, the literature examining confidence biases and personality traits together has found scant evidence for their potential relationship, and generally focuses on over-confidence (Moore and Schatz, 2017).

Suggesting that some of the variance in self-assessment accuracy has its source in stable individual differences (Stankov and Crawford, 1996, 1997; Kleitman and Stankov, 2001, 2007; Pallier et al., 2002; Campbell et al., 2004; Jain and Bearden, 2011; Buratti et al., 2013; Kasperski and Katzir, 2013; Burns et al., 2016), this research examines the interaction between accuracy on cognitive tests and confidence in the same, and the predictive value of this interaction for a broad personality questionnaire commonly employed in career development settings (Gough and Bradley, 2005). Establishing a link between the decision-making styles reflected in confidence biases and personality traits has value for designing employee development programs that are sensitive to the dispositions of the people who need them (Rebele et al., 2021). Addressing the default decision-making styles of employees will be supported by understanding the traits that co-occur with under- vs. over-confidence, respectively. This study is based on the following research question:

*RQ.* Do discrepancies in ability and confidence in either direction predict individual differences on a broad-spectrum and commercially available personality test, the California Psychological Inventory—260 (CPI260; Gough and Bradley, 2005)?

We start by reviewing the theoretical accounts of confidence bias and the major advancements linking it to individual differences. We then examine the potential relationships between confidence bias and the CPI260, based on previous literature examining associations between the CPI260 and five-factor model personality traits (Deniston and Ramanaiah, 1993; McCrae et al., 1993; Fleenor and Eastman, 1997; Gough and Bradley, 2005).

### 1.1. Theory and hypotheses

Theoretical accounts of confidence bias differ on whether errors in self-assessment result from the procedures involved in the creation and structure of cognitive test items, or whether they arise from biases present within the individual. Proponents of an ecological approach to confidence bias suggest that the cues to accurately solving a problem reside within the items on the task (Gigerenzer et al., 1991). In support of the ecological approach, confidence items do appear to be sensitive to the nature of the task and the difficulty of task items (Olsson and Winman, 1996; Stankov and Crawford, 1996, 1997; Kleitman and Stankov, 2001). In contrast to the ecological approach, a heuristics and biases account of confidence suggests that general cognitive biases, mental shortcuts, or both are involved in predictions about accuracy on a task (Tversky and Kahneman, 1974). These mental shortcuts reside within the individual, and provide a technique for rapid problem solving that might lead to characteristic errors that create miscalibration between accuracy and confidence (Jackson et al., 2016). Generally the focus of experimental work on confidence has been on the cognitive processes across individuals that produce over-confidence specifically (Parker and Fischhoff, 2005).

A dispositional approach to confidence bias suggests that some individuals might be more over-confident, while others display more under-confidence. Linking confidence biases with personality traits would give us some insight into the characteristics of individuals who are high vs. low on the confidence factor. Consensus on the structure of personality traits has generally converged around the five-factor model, a factor-analytic structure consisting of five characteristics labeled Agreeableness, Conscientiousness, Extraversion, Neuroticism, and Openness to Experience (McCrae and Costa, 1997). The interpersonal traits representing positive affect include Agreeableness (A) and Extraversion (E). Agreeableness describes a cooperative, trusting, and sympathetic individual, where Extraversion reflects individuals high on social activity with a general tendency to experience positive emotions. Trait emotional negativity is represented by Neuroticism, the tendency to experience negative emotions and psychological distress. The Conscientiousness dimension describes a tendency to be hard working, diligent, and well-organized, while Openness to Experience, the final trait, describes the tendency to be open to new ideas and experiences (Costa and McCrae, 1992). The five-factor model tends to replicate across cultures (Costa et al., 2001; Paunonen et al., 2003; McCrae et al., 2005), can be located on the human genome (Jang et al., 1998; Yamagata et al., 2006), and predict life outcomes including health (Ozer and Benet-Martinez, 2006; Roberts et al., 2007), relationship quality (Roberts et al., 2007; Malouff et al., 2010), job performance (Barrick et al., 2001), and income (Sutin et al., 2009).

Research linking personality traits with confidence bias has produced mixed results. Using the five-factor model as our organizing model of personality (Costa and McCrae, 1992), over-confidence appears to be associated with Extraversion (E; Schaefer et al., 2004), Agreeableness (A; Sukenik et al., 2018; Mayer et al., 2020), and lower Neuroticism (N; Mayer et al., 2020). Those higher in trait Narcissism (Campbell et al., 2004) were also more likely to show over-confidence, potentially suggesting higher scores on indices of Extraversion due to a shared relationship between Narcissism and E (Paulhus and Williams, 2002). Higher scores on Narcissism and Machiavellianism (Jain and Bearden, 2011) further implicate lower scores on Conscientiousness and Agreeableness, respectively, (O’Boyle et al., 2015). From these results and based on a linear understanding of confidence bias, we might infer under-confident individuals are more introverted (Low E), and higher on measures reflecting Neuroticism (N). In support of this inference, Stone et al. (2001) examined under-confidence in the context of depression. They found that depressed individuals demonstrated under-confidence in their aggregate performance judgments. Depression itself has been linked with Neuroticism and introverted tendencies (Jourdy and Petot, 2017; Lyon et al., 2021). This suggests that we might expect over-confident individuals to show general tendencies toward more positive affect, while those who are under-confident might show a propensity toward emotional instability, negative affect, and more introverted characteristics.

A broad measure of personality is ideal for an exploratory study of this nature, in contrast to targeted measures that might be more appropriate for confirmatory research. The California Psychological Inventory–260 (CPI260; Gough and Bradley, 2005) assessment was designed to provide deep and complex personality insights for recruitment and development purposes. The CPI260 measure includes 29 scales across five categories, comprising individuals’ capacity to deal with others (seven scales), to manage themselves (seven scales), their motivations and thinking styles (three scales), their personal characteristics (three scales), and work-related measures (six scales). Further, the CPI260 has demonstrated associations with measures of career advancement that have also been associated with indices of confidence (Schaubhut et al., 2011). Because the CPI260 has not been modeled with confidence before, we made only tentative predictions on the likely relationships between confidence bias and CPI260 scales based on the associations between CPI scales and five factor model personality traits found in previous literature. The CPI factors are best described by composites of five-factor model traits, which makes straightforward interpretation of associations difficult (Soto and John, 2009). We nonetheless used the five-factor model associations with the CPI measures to guide our exploration of confidence bias effects (Deniston and Ramanaiah, 1993; McCrae et al., 1993; Fleenor and Eastman, 1997; Gough and Bradley, 2005). Any hypotheses we posed below are therefore only tentative expectations.

#### 1.1.1. Positive affect and over-confidence

Over-confidence might be associated with specific subscales of the CPI260 that reflect trait positive affectivity, including Extraversion and Agreeableness. The *Dealing with Others* scales, including Dominance (Do), Capacity for Status (Cs), Sociability (Sy), Social Presence (Sp), Self-Acceptance (Sa), Independence (In), and Empathy (Em) have been consistently and positively associated with five factor model Extraversion (Deniston and Ramanaiah, 1993; McCrae et al., 1993; Fleenor and Eastman, 1997; Gough and Bradley, 2005). In contrast to Extraversion, Agreeableness has been implicated in both over- and under-confidence (Campbell et al., 2004; Jain and Bearden, 2011; Sukenik et al., 2018), and is not as well represented by the California Psychological Inventory (Deniston and Ramanaiah, 1993; Soto and John, 2009). Ratings of CPI items by experts on the five factor model did not identify strong links between the CPI scales and trait Agreeableness, however, a follow-up study by the same authors examining simple correlations between the five factor model and CPI scales in a community sample found a positive association between Agreeableness and the *Personal Characteristic* of Sensitivity (Sn; McCrae et al., 1993). In contrast, Fleenor and Eastman (1997) identified the *Self-Management* scale Communality (Cm) as a positive correlate of trait Agreeableness. Correlations between CPI260 scales and five factor model traits reported in the CPI260 manual suggested positive associations between Agreeableness and the *Self-Management* scales of Self-Control (Sc) and Good Impression (Gi; Gough and Bradley, 2005). The more recently developed *Work-Related Measures* of the CPI260 also showed positive associations between Agreeableness and Amicability (Ami; Gough and Bradley, 2005).

H1. We expected over-confidence to predict increased scores on the *Dealing with Others* scales of the CPI260. Conversely, as individuals became more under-confident, we expected decreased scores on the *Dealing with Others* scales of the CPI260.

H2. In line with possible associations between Agreeableness and confidence bias in both directions, we expected increased scores on *Self-Management* scales of Self-Control (Sc), Good Impression (Gi) and Communality (Cm) for both over- and under-confident participants. We also anticipated increased scores for both under- and over-confident participants on the *Personal Characteristics* construct of Sensitivity (Sn), and the *Work-Related Measure* of Amicability (Ami).

#### 1.1.2. Negative affect and under-confidence

Lower Neuroticism has also been implicated in over-confidence (Mayer et al., 2020) and higher Neuroticism with under-confidence. The *Dealing with Others* scale Independence (In) was negatively associated with Neuroticism across all studies and samples we identified, with the *Personal Characteristics* scale Sensitivity (Sn) positively related across all samples (Deniston and Ramanaiah, 1993; McCrae et al., 1993; Fleenor and Eastman, 1997; Gough and Bradley, 2005). Lower scores on the *Self-Management* scales of Self-Control (Sc) and Good Impression (Gi) were also implicated in higher Neuroticism. The *Personal Characteristics* constructs of Wellbeing (Wb) and Insightfulness (Is) were negatively associated with Neuroticism across two samples (McCrae et al., 1993; Gough and Bradley, 2005). Neuroticism was also inconsistently related to lower *Motivations and Thinking Styles*, with lower Achievement via Independence (Ai) potential (Gough and Bradley, 2005) lower Achievement via Conformance (Ac; McCrae et al., 1993), and a negative relationship between Conceptual Fluency (Cf) and Neuroticism observed (McCrae et al., 1993; Gough and Bradley, 2005).

H3. As accuracy decreases and confidence increases, we expect to see scores on CPI260 scales reflecting lower Neuroticism, particularly higher scores on *Dealing with Others* scale Independence (In), *Self-management* scales of Self-Control (Sc), Good Impression (Gi), and Wellbeing (Wb), the *Personal Characteristics* scale of Insightfulness (Is), and lower *Motivations and Thinking Styles* including Achievement via Conformance (Ac), Achievement via Independence (Ai), and Conceptual Fluency (Cf) scores. Lower scores on the *Personal Characteristics* scale Sensitivity (Sn) were also expected in those who were over-confident. As accuracy increased and confidence decreased, we anticipated lower scores on the same scales, with higher scores on Sensitivity (Sn).

#### 1.1.3. Poorer self-management and over-confidence

Conscientiousness was only indirectly implicated in confidence bias effects through its shared association with Dark Triad personality traits (Paulhus and Williams, 2002; O’Boyle et al., 2015). Over-confidence should be associated with lower trait Conscientiousness, with the work of Jackson and Kleitman (2014) and Jackson et al. (2016, 2017) suggesting that under-confident individuals might show a tendency to make more considered decisions, a behavior reflective of higher trait Conscientiousness. Increased Conscientiousness has also been recently associated with accurate decision-making in the context of high ability (Mayer et al., 2020). Both Fleenor and Eastman (1997) and Gough and Bradley (2005) suggest positive relationships between Conscientiousness and CPI *Self-Management* constructs, including Responsibility (Re), Social Conformity (So), Self-Control (Sc), Good Impression (Gi), and Wellbeing (Wb). The *Motivations and Thinking Style* scale Achievement via Conformance (Ac) was also positively associated with Conscientiousness across all four studies, as was a negative association with the *Personal Characteristic* of Flexibility (Fx; Deniston and Ramanaiah, 1993; McCrae et al., 1993; Fleenor and Eastman, 1997; Gough and Bradley, 2005). Gough and Bradley (2005) also found positive associations between Conscientiousness and *Work-Related Measures* of Work Orientation (Wo) and Leadership Potential (Lp).

H4. We expected over-confidence in the form of increased confidence and decreased accuracy to be associated with lower scores on *Self-Management* scales of the CPI260 including Responsibility (Re), Social Conformity (So), Self-Control (Sc), Good Impression (Gi), and Wellbeing (Wb), as well as lower scores on Achievement via Conformance (Ac), and higher scores on Flexibility (Fx). Conversely, under-confidence should predict higher scores on these CPI260 scales.

H5. In line with the findings of Mayer et al. (2020), we further expected a match between accuracy and confidence at high accuracy levels to be associated with CPI260 traits reflecting higher trait Conscientiousness.

#### 1.1.4. Openness and confidence

Finally, Openness has been implicated in accurate decision-making as suggested by a match between higher accuracy and matching confidence (Mayer et al., 2020). The *Motivations and Thinking Style* scale Achievement via Independence (Ai) was most often positively associated with Openness measures (Deniston and Ramanaiah, 1993; McCrae et al., 1993; Fleenor and Eastman, 1997; Gough and Bradley, 2005), followed by Conceptual Fluency (Cf; McCrae et al., 1993; Fleenor and Eastman, 1997; Gough and Bradley, 2005). The *Personal Characteristics* Insightfulness (Is) and Flexibility (Fx) were also positively associated with Openness across most of the studies we identified (Deniston and Ramanaiah, 1993; McCrae et al., 1993; Fleenor and Eastman, 1997; Gough and Bradley, 2005). The *Self-Management* scale Tolerance (To) positively loaded onto an Openness factor in the work of Fleenor and Eastman (1997), while McCrae et al. (1993) found positive associations between Openness and five *Dealing with Others* scales including Capacity for Status (Cs), Social Presence (Sp), Self-Acceptance (Sa), Independence (In), and Empathy (Em).

H6. As accuracy and confidence increased together, we expected to see increases in personality traits suggesting higher Openness, including *Motivations and Thinking Style* constructs of Achievement via Independence (Ai) and Conceptual Fluency (Cf), the *Personal Characteristics* of Insightfulness (Is) and Flexibility (Fx), the *Self-Management* scale Tolerance (To), and the *Dealing with Others* scales of Capacity for Status (Cs), Social Presence (Sp), Self-Acceptance (Sa), Independence (In), and Empathy (Em).

Inspecting the associations between CPI260 scales and the five factor model personality traits led us to some contradictory predictions for the association between CPI260 scales and confidence bias. For example, based on an association between Self-Control (Sc) and Neuroticism, we predicted that over-confident individuals would be high on Self-Control (Sc) while under-confident individuals would be low on the same characteristic. However, an association between Self-Control (In) and Conscientiousness led us to expect lower Self-Control in over-confident participants, and higher self-control in those who were under-confident. We identified contradictory expectations for four CPI260 scales, including Self-Control (Sc), Good Impression (Gi), Wellbeing (Wb), and Achievement via Conformance (Ac). In these cases, we examined the results for each of these scales to determine whether the pattern we observed matched any of our expectations.

## 2. Materials and methods

### 2.1. Participants

This study was a retrospective analysis of data already collected as a part of commercial operations between Consulting Psychologists Press and ebilities Pty Ltd. Participants were 225 employed adults (39.1% male, *N* = 88) from the USA (51.6%, *N* = 116) and Australia (48.4%, *N* = 109) who had previously taken the CPI260 assessment, and who were asked to take the ebilities cognitive test suite described below. The sample had a mean age of 44.43 years (*SD* = 11.23). Five participants were removed from analysis due to not reporting age or gender. Two-hundred and twenty participants remained in subsequent analyses. Participants were known to Consulting Psychologists Press in both the USA and Australia, but anonymous in all respects to both ebilities Pty Ltd and the research team, therefore further demographic data was not available.

### 2.2. Measures

#### 2.2.1. Accuracy

The ebilities General Mental Ability—3 (GMA-3; Douglas and Cadman, 2021) battery was designed to provide three core tests of cognitive abilities: Swaps, Vocabulary, and Numerical Operations. These tests measure some of the key cognitive abilities described by the theory of fluid and crystallized intelligence (Horn and Cattell, 1966). All items included within the battery had cut-off times. Scores from each of the three core tests were combined into percentage accuracy, to ensure the cognitive measure was reported in the same scale as the Confidence measure described below.

Test of Fluid Ability (Gf)–Swaps. This was a test of fluid ability that involved working memory. Test-takers were shown a set of three pictures and were given an instruction about swapping the order of the pictures, for example, “Swap 2 and 3.” They were then shown an answer screen, which included the same three pictures in six different orders. Participants were asked to select the option that presented the correct sequence of pictures after the swap had been made. Test items ranged between 1 and 4 swaps, with item complexity increasing as more swaps were required. There were 20 items on this test. The internal reliability of the Swaps test in this sample was α = 0.83.

Test of Crystallized Ability (Gc)–Vocabulary. This was a test of word knowledge that measured crystallized ability. A word was displayed on the screen and four possible synonyms were shown below it. Test-takers were instructed to select the word that meant the same as the target word from among the four options. This test consisted of 30 items that varied in difficulty. The internal reliability on this test was α = 0.77.

Test of Quantitative Knowledge (Gq)–Numerical Operations. This test consisted of mathematical questions that requested the participants to solve by using addition, subtraction, division, and multiplication, and select the correct solution to the problem from the four possible options below it. There were 25 items in the test that varied in difficulty and were completed without the use of a calculator. The Numerical Operations test had an internal reliability of α = 0.84.

#### 2.2.2. Confidence

Confidence was measured by embedding survey questions into each of the cognitive ability tests. After each test item, participants were asked to rate how confident they were that they answered the preceding question correctly. The response options ranged between the chance of guessing the correct answer (for example 25% for a four-option answer format) and 100% confidence. Consistent with the evidence indicating that Confidence forms a single factor regardless of the cognitive test it is yoked to (Stankov and Crawford, 1996, 1997; Stankov, 2000; Stankov and Lee, 2008; Stankov et al., 2013), an average Confidence score was calculated across all three tests. The internal reliability of the Confidence composite was α = 0.98.

#### 2.2.3. Personality

The California Psychological Inventory (CPI260; Gough and Bradley, 2005). All 29 scales in the CPI 260 instrument were derived from their longer counterparts in the standard 434 item version of the CPI instrument and correlate highly with the longer version of the scale, *r*s = 0.94 to 0.95. The CPI260 manual also reports median internal consistency reliability across three separate samples between 0.70 and 0.76, median test–retest correlations over 1 year as 0.66, and over 10 years as 0.77 (Gough and Bradley, 2005). All scale scores are reported as *T*-scores, with a mean of 50 and a standard deviation of 10. CPP provided the research team with composite scores for the CPI260, therefore we were unable to calculate internal reliabilities for this sample.

### 2.3. Procedure

Participants were provided with a login to complete the ebilities GMA-3 assessments online as part of a research and development project between Consulting Psychologist’s Press (CPP) and ebilities Pty Ltd. Participants who had already completed the CPI260 were invited by CPP to complete the ebilities GMA-3 assessments if they wished. The ebilities results were not linked to any high stakes testing procedure. Together, the test and questionnaires took approximately 90 min to complete, and participants were informed that they could withdraw their data from any research project on the ebilities online testing platform. Ethical approval was obtained to analyze non-identifiable data after it was collected (HREC H-2020−0237).

### 2.4. Statistical analysis

Previous analysis indicated limited differences in the measurement scales between USA and Australian samples (Douglas and Cadman, 2021), therefore we analyzed all participants as a single group. Data analysis involved multiple sequential steps that were performed in R (Version 4.1.1; R Core Team, 2021). Data were first screened for missing values and violations of the assumptions of multiple regression as per the advice of Tabachnick and Fidell (2013). Polynomial regression with response surface analysis was then used to model both matches and discrepancies between the GMA-3 accuracy and confidence measures (Shanock et al., 2010; Barranti et al., 2017). Interested readers can find the published tutorials we used to analyze our data in both Shanock et al. (2010) and Barranti et al. (2017). Polynomial regression with response surface analysis (RSA) assesses whether matches and mismatches matter by modeling the consequences of all possible combinations of two predictors for an outcome (Barranti et al., 2017). The analysis approach represents an extension of a general multiple regression that incorporates squared and cross-product terms, allowing researchers to explicitly test a larger range of both linear and non-linear hypotheses (Nestler et al., 2015). Further, all relevant variables are preserved in their original form without using mathematical operations that conceal or distort information (Nestler et al., 2015; Barranti et al., 2017), thereby overcoming the issues with difference and residual scores used in prior literature. It extends the researcher’s capacity to make inferences about matches in general and allows more specific testing at different levels of each predictor. Rather than testing a general question about whether a match is better or worse than a mismatch, RSA allows us to model how specific matches and mismatches are uniquely predictive of personality traits. RSA further provides a thorough visualization of any observed effects through 3-D response surface plots, a feature which facilitates understanding and interpretation of the results (Barranti et al., 2017).

We used the “*RSA*” package for R to conduct our polynomial tests (Schönbrodt and Humberg, 2021). Consistent with the polynomial regression technique, each of the CPI260 subscales were regressed on percentage accuracy, confidence and their squared and cross-product terms (Edwards, 2002). Following Shanock et al. (2010), we first examined the percentage accuracy and confidence variables to determine the proportion of participants with discrepancies between the two in either direction. Age and gender were controlled where they were significant predictors of the dependent variable because previous research has suggested both age and gender are associated with over- and under-confidence effects (Pallier, 2003; Burns et al., 2016). A significant change in r-squared (Δ*R*^2^) associated with the cross-product and squared terms was used to justify the interpretation of surface values.

If Δ*R*^2^ was significantly greater than zero, the model coefficients were transformed into five surface test values: *a*_1_ to *a*_5_ (Edwards and Parry, 1993; Shanock et al., 2010; Schönbrodt et al., 2018). The line of agreement was assessed by surface tests *a*_1_ and *a*_2_, and describes where accuracy and confidence match. This line runs from the front to the back corner of every figure. The values of *a*_1_ (b_*GMA*_ + b_*Con*_) test the linear relationship between a perfect match and the personality scale. Surface test *a*_1_ assesses whether agreement between accuracy and confidence has a different effect on personality traits at higher or lower levels of the ability/confidence scale and corresponds with hypotheses 5 and 6. Support for these two hypotheses would be indicated by a significant and positive *a*_1_ for personality traits reflecting Conscientiousness and Openness, respectively. In contrast, *a*_2_ (b^2^_*GMA*_ + b_GMA*Con_ + b^2^_*Con*_) reflects the non-linear relationship between the agreement in accuracy and confidence scores and the personality outcome and helps us to answer whether matches at extremes have different personality profiles to matches at midrange levels. We did not make any predictions associated with surface test *a*_2_.

The line of disagreement, where accuracy increases as confidence decreases, runs from the left corner to the right corner of the graph. Surface tests *a*_3_ and *a*_4_ are associated with the line of disagreement, where a_3_ assessed the slope and *a*_4_ assessed the curve of that line. The values of *a*_3_ (b_*GMA*_–b_*Con*_) reveal whether the direction of a discrepancy between accuracy and confidence matter by testing the slope of the line of disagreement. Tests of hypotheses 1, 3, and 4 rely on an inspection of surface test *a*_3_. A positive *a*_3_ value suggests that personality scores increase as accuracy becomes greater than confidence (i.e., under-confidence). Negative *a*_3_ values indicate personality traits that increase as individuals become more over-confident. The values for *a*_4_ (b^2^_GMA_−b_GMA*Con_ + b^2^_Con_) indicate whether personality traits become more extreme in either direction as accuracy and confidence diverge (Shanock et al., 2010). Surface test *a*_4_ provides a test of whether mismatches matter overall, an effect we predicted for Agreeableness-related CPI260 scales in hypothesis 2.

Negative coefficients on the *a*_2_ and *a*_4_ surface tests indicate concave patterns along their respective lines. For example with *a*_2_, this indicates that personality trait scores are decreasing as matches between ability and confidence become more extreme in either direction. Positive coefficients for *a*_2_ and *a*_4_ indicate convex curves, where scores on the outcome variables increase. For *a*_4_, this indicates that scores on personality traits are becoming higher as mismatches become more extreme in either direction. Finally, *a*_5_ (b^2^_GMA_–b^2^_Con_) tests the ridge line of the response surface to determine whether it is positioned exactly on the line of agreement. Values closer to zero indicate that the ridge line of the surface is positioned closer to the line of agreement.

## 3. Results

Data were screened and assumptions of normality, linearity, and homoscedasticity were satisfied (Tabachnick and Fidell, 2013). Therefore, parametric tests were deemed appropriate in all cases. Shanock et al. (2010) recommends centering both predictors around the midpoints of their respective scales to aid interpretation and reduce issues with multicollinearity (Aiken and West, 1991). Therefore, accuracy and confidence were centered on a scale midpoint of 75, a value which reflected a compromise between the numerical scale midpoint of 62.5 (halfway between the chance of guessing and perfect accuracy), and the mean value on both accuracy and confidence above 80 (see Table 1). Data were further inspected to ensure discrepancies in accuracy and confidence values were present in our sample. Preliminary checks indicated that 41.8% of participants (*N* = 225) had no discrepancy between their accuracy and confidence. Participants with greater confidence than their accuracy (over-confidence) made up 28.9% of our sample and participants with less confidence than their accuracy (under-confidence) made up 29.3% of our sample. All assumptions for polynomial regression were satisfied. Five participants were removed from analysis due to not reporting age or gender. Two-hundred and twenty participants remained in subsequent analyses.

**TABLE 1:** Correlations between confidence and the CPI260 scales.

	Mean	SD	*r* Accuracy	*r* Confidence
Age	44.43	11.23	0.19	0.25
**GMA3**				
Accuracy	82.24	9.08	-	
Confidence	86.22	9.30	0.47*	-
**California psychological inventory**				
**Dealing with others**				
Dominance (Do)	58.89	9.04	0.03	0.11
Capacity for status (Cs)	57.99	8.79	0.04	0.14
Sociability (Sy)	54.47	9.93	-0.06	0.09
Social presence (Sp)	54.03	10.48	0.05	0.06
Self-acceptance (Sa)	57.44	9.42	-0.07	0.01
Independence (In)	59.35	8.73	0.08	0.16
Empathy (Em)	61.53	9.42	0.07	0.11
**Self-management**				
Responsibility (Re)	55.58	7.09	0.20*	0.16
Social conformity (So)	52.22	8.21	0.07	0.07
Self-control (Sc)	54.63	9.21	0.03	0.00
Good impression (Gi)	56.11	8.45	-0.03	0.04
Communality (Cm)	50.39	7.79	0.05	0.10
Wellbeing (Wb)	53.38	8.42	0.02	0.10
Tolerance (To)	58.64	7.80	0.15	0.08
**Motivations and thinking style**				
Achievement via conformance (Ac)	55.81	6.89	0.14	0.14
Achievement via independence (Ai)	60.83	7.03	0.21*	0.05
Conceptual fluency (Cf)	56.84	7.36	0.19	0.20*
**Personal characteristics**				
Insightfulness (Is)	56.29	7.93	0.05	0.12
Flexibility (Fx)	56.43	11.18	0.12	0.05
Sensitivity (Sn)	47.23	8.41	-0.08	-0.11
**Work-related measures**				
Managerial potential (Mp)	62.07	7.84	0.14	0.15
Work orientation (Wo)	54.30	7.71	0.17	0.17
Creative temperament (Ct)	58.53	10.55	0.10	0.11
Leadership potential (Lp)	59.57	8.25	0.04	0.11
Amicability (Ami)	55.04	9.19	0.07	0.11
Law enforcement orientation (Leo)	55.37	9.24	-0.01	−0.02

All significance tests were holm-adjusted (Gaetano, 2018). *p < 0.001.

### 3.1. Descriptive statistics and correlations

Descriptive statistics and correlations between the CPI260 scales and GMA-3 overall accuracy and confidence measures can be found in Table 1. Accuracy on the cognitive tests was associated with confidence in the same tests. Accuracy on the tests was also significantly and positively associated with Responsibility (Re) and Achievement via Independence (Ai). This suggested that those with higher accuracy on the cognitive test were also more likely to comply with societal rules as appropriate and have higher achievement potential in open situations where creativity and proactivity are required for success. Confidence was positively and significantly associated with Conceptual Fluency (Cf). This suggested that those with higher confidence in their cognitive test answers had more belief in their own ability to deal with abstract and complex concepts.

### 3.2. Polynomial regressions

The results of the polynomial regression analyses can be found in Table 2. The polynomial regression variables significantly predicted Social Presence (Sp), Communality (Cm), Wellbeing (Wb), Achievement via Conformance (Ac), Achievement via Independence (Ai), Insightfulness (Is), and Leadership Potential (Lp). Although the findings for Wb, Is, and Lp were significant overall, and *a*_4_ was significant in all three models, the size of the effect was so small that the *z*-axis only covered one *T*-score point. We therefore did not interpret these models, but they were included in Table 2 for reference.

**TABLE 2:** Significant polynomial regression and response surface analysis tests for CPI260.

Variable	Social presence	Communality	Wellbeing	Achievement via conformance	Achievement via independence	Insightfulness	Leadership potential
	**b(se)**	**b(se)**	**b(se)**	**b(se)**	**b(se)**	**b(se)**	**b(se)**
Constant	55.04 (1.23)	50.86 (0.90)	54.70 (0.93)	55.48 (0.80)	61.18 (0.82)	56.31 (0.94)	60.43 (0.95)
**Demographic controls**							
Gender	–	–	–	–	–	–	–
Age	–	–	0.17 (0.05)	–	–	0.06 (0.05)	0.17 (0.05)
**Polynomial coefficients**							
Accuracy	1.59 (1.01)	−0.93 (0.83)	−0.10 (0.09)	0.32 (0.66)	0.76 (0.65)	−0.13 (0.09)	−0.03 (0.09)
Confidence	−0.17 (1.06)	−2.02 (0.85)	−0.03 (0.10)	−1.35 (0.62)	−0.46 (0.54)	−0.06 (0.10)	−0.04 (0.10)
Accuracy^2^	−0.02 (0.01)	−0.02 (0.01)	−0.02 (0.01)	−0.01 (0.00)	−0.01 (0.00)	−0.01 (0.01)	−0.02 (0.01)
Accuracy × Confidence	0.02 (0.01)	0.02 (0.01)	0.02 (0.01)	0.01 (0.01)	0.01 (0.01)	0.02 (0.01)	0.01 (0.01)
Confidence^2^	−0.01 (0.01)	0.00 (0.01)	0.00 (0.01)	0.00 (0.00)	0.00 (0.00)	0.00 (0.01)	0.00 (0.01)
**Omnibus statistics**							
*F* (df)	3.70 (5, 214)	5.55 (5, 214)	6.22 (6, 213)	2.76 (5, 214)	3.18 (5, 214)	3.90 (3, 213)	3.15 (3, 213)
*p*	0.013	0.001	<0.001	0.037	0.025	0.010	0.026
Δ*R*^2^	0.04*	0.06**	0.07***	0.02*	0.03*	0.05**	0.04*
**Response surface tests**							
*a*_1_	1.42 (1.23)	−1.09 (0.98)	−0.14 (0.11)	−1.03 (0.82)	0.30 (0.68)	−0.19 (0.11)	−0.07 (0.12)
*a*_2_	−0.01 (0.01)	0.01 (0.01)	0.00 (0.01)	0.01 (0.01)	0.00 (0.00)	0.01 (0.01)	0.00 (0.01)
*a*_3_	1.75 (0.01)	2.96 (1.38)*	−0.07 (0.15)	1.67 (0.98)	1.22 (0.97)	−0.07 (0.15)	0.01 (0.15)
*a*_4_	−0.05 (0.01)**	−0.03 (0.01)**	−0.04 (0.01)*	−0.02 (0.01)*	−0.03 (0.01)**	−0.02 (0.01)*	−0.02 (0.01)*
*a*_5_	−0.01 (0.01)	−0.02 (0.01)	−0.02 (0.01)	−0.01 (0.01)	−0.01 (0.01)	−0.01 (0.01)	−0.02 (0.01)

*p < 0.05. **p < 0.01. ***p < 0.001. b = regression coefficient; se = standard error; Δ*R*^2^ = change in r-squared associated with squared and cross-product predictors; a_1_ and a_2_ refer to slope and curve along the line of agreement, respectively; a_3_ and a_4_ refer to the slope and curve along the line of disagreement, respectively.

**FIGURE 1:**
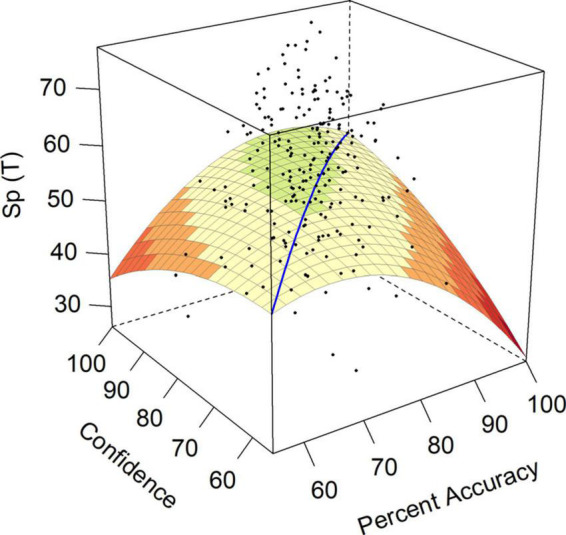
Response surface for social presence (Sp).

Social Presence (Sp) was implicated in both hypotheses 1 and 6. Hypothesis 1 suggested that over-confidence should predict an increase in Social Presence (Sp), as reflected in a negative *a*_3_ surface test. Hypothesis 6 suggested we should see increased Social Presence (Sp) as ability and confidence increased together, as demonstrated by a positive and significant *a*_1_ surface test. In contrast to our expectations in either hypothesis, surface test *a*_4_ for Social Presence (Sp) was significant and negative, indicating that a mismatch between accuracy and confidence in either direction predicted decreased Social Presence scores. The surface model for Social Presence is shown in Figure 1. While over-confident individuals had a *T*-score on Social Presence of approximately 35, under-confident individuals had even lower *T*-scores of 25. Both were significantly below the normative mean for this construct and well below the overall sample mean of 54.03 (see Table 1).

**FIGURE 2:**
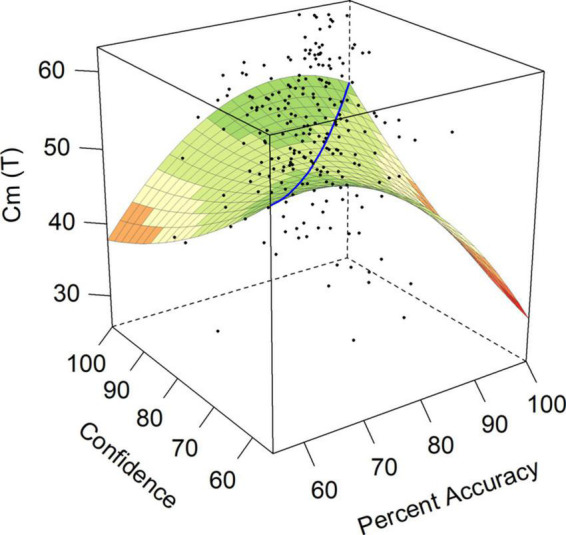
Response surface for communality (Cm).

Communality (Cm) was addressed in hypothesis 2, where we expected a significant and positive *a*_4_ surface test. Instead, the model for Communality had significant negative surface tests *a*_3_ and *a*_4_, indicating that the line of disagreement between accuracy and confidence had a non-linear relationship with this personality trait, so that increasing discrepancies in either direction led to lower scores on Communality. The surface model for Communality can be found in Figure 2. Participants who were both under-confident and over-confident, as indicated by surface test *a*_4_, had lower Communality scores than participants with average and matching levels of accuracy and confidence (*T* = 47). Communality also had a significant negative *a*_3_, indicating that Communality scores were higher when the participant was over-confident (*T* = 38) as compared to under-confident (*T* = 32).

**FIGURE 3:**
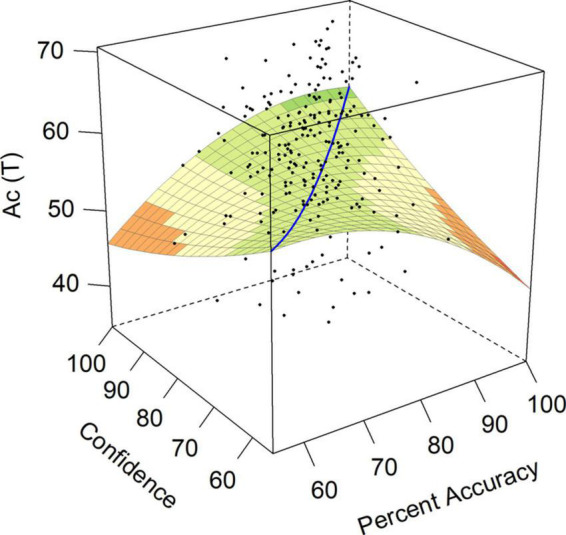
Response surface for achievement via conformance (Ac).

Achievement via Conformance (Ac) was relevant to hypotheses 3, 4, and 5. Ac was associated with lower Neuroticism, suggesting that over-confident individuals should be higher in Ac (hypothesis 3), and we should observe a negative *a*_3_ surface test. In contrast, Ac was also associated with higher Conscientiousness, suggesting that *under-confident* individuals should be higher in Ac (hypothesis 4), which corresponds with a positive *a*_3_ surface test. Hypothesis 5 suggested that we might expect higher Ac when accuracy and confidence were both high, as indicated by a significant and positive *a*_1_ surface test. The surface model for Achievement *via* Conformance is presented in Figure 3. For Achievement by Conformance, surface test *a*_4_ was significant and negative (see Table 2), indicating a negative curvature along the line of disagreement. Unexpectedly, this indicated that as individuals became more over-confident and more under-confident, their self-rated ability to achieve under structured situations decreased.

**FIGURE 4:**
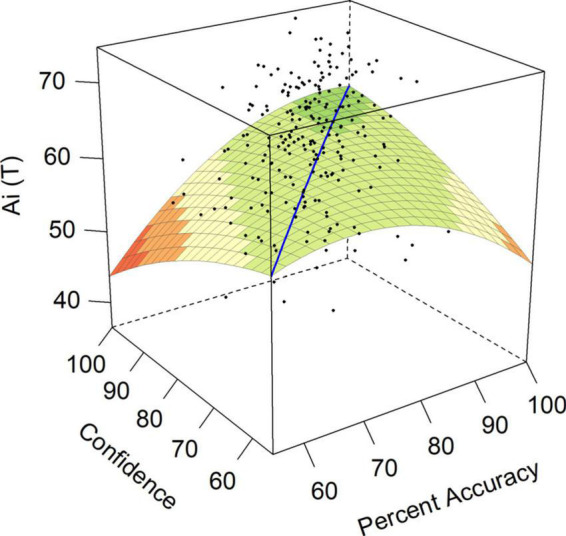
Response surface for achievement via independence.

Achievement via Independence (Ai) was relevant to both hypotheses 3 and 6. Hypothesis 3 suggested a negative *a*_3_ surface test indicating increased Ai scores when individuals were over-confident. Hypothesis 6 would be supported by a positive *a*_1_, indicating increased Ai scores as ability and confidence increased together. As shown in Table 2, surface test *a*_4_ for Ai was significant and negative, indicating that as accuracy and confidence diverged in either direction, the personality traits reflecting ability to achieve in unstructured situations decreased. Figure 4 demonstrated this concave curve where over-confident individuals and under-confident individuals had Achievement via Independence scores over 1 SD below average accuracy/confidence individuals in this sample.

## 4. Discussion

We set out to explore the possible association between confidence bias and personality traits in a sample of working adults who had completed both an accuracy measure with yoked confidence items, and the CPI260, a personality tool commonly employed in workplace settings (Gough and Bradley, 2005). We developed tentative hypotheses on the relationships between over-confidence, under-confidence, and matching ability and confidence levels for CPI260 personality traits. These were based on previous research with confidence metrics, five-factor model personality traits, and their shared association with CPI personality constructs (Deniston and Ramanaiah, 1993; McCrae et al., 1993; Fleenor and Eastman, 1997; Gough and Bradley, 2005). To the best of our knowledge, this is the first study to integrate an examination of over- and under-confidence in a working adult population using a technique that allows for the modeling of non-linear relationships between confidence bias and personality traits (Shanock et al., 2010; Nestler et al., 2015; Barranti et al., 2017). The research contains implications for researchers and workplace practitioners alike.

The results of this study did not align with previous literature. Only four CPI260 constructs showed evidence of significant polynomial terms that had meaningful variation, and in every instance these results defied our expectations. We found significant and negative *a*_4_ surface tests across four traits, indicating that the mismatch between ability and confidence more generally predicted decreased scores on Social Presence (Sp), Communality (Cm), Achievement via Conformance (Ac), and Achievement via Independence (Ai). In contrast to previous literature identifying traits for either over-confident (Campbell et al., 2004; Schaefer et al., 2004; Jain and Bearden, 2011; Sukenik et al., 2018; Mayer et al., 2020) or under-confident individuals (Stone et al., 2001), over-confident individuals in our study were more like those who were under-confident. Previous literature has focused on over-confidence (Campbell et al., 2004; Schaefer et al., 2004; Jain and Bearden, 2011). By omitting an explicit treatment of under-confidence, these previous studies have implied that the higher scores on the dispositions that are associated with over-confidence are conversely low in the under-confident. Our study has subjected this implicit assumption to empirical testing and has revealed that it might be false. The failure of previous research to identify such effects is likely a function of the use of difference scores to calculate bias (Stone et al., 2001; Pallier et al., 2002; Campbell et al., 2004; Schaefer et al., 2004; Jain and Bearden, 2011; Moore and Dev, 2018; Sukenik et al., 2018; Mayer et al., 2020). The findings of this study tentatively support this possibility because we did identify significant non-linear effects along the line of disagreement for four CPI260 scales using a different modeling tool.

Both over- and under-confident employees in our sample had lower scores on measures of achievement potential that have been associated with success in organizational settings (Gough and Bradley, 2005). Somewhat unexpectedly, we found that the achievement measures on the CPI260, Achievement via Conformance (Ac) and Achievement via Independence (Ai), were negatively associated with increasing discrepancies between accuracy and confidence in either direction. In other words, both those who were under-confident and those who were over-confident had lower scores on these two personality characteristics. These findings suggest that the default personality setting of over- and under-confident individuals is to doubt their achievement potential. They also offered partial support for hypothesis 3, such that we only predicted decreased scores on Ac and Ai for under-confident participants reflecting their increased Neuroticism (Stone et al., 2001). The finding of a decreased score on Ac for over-confident individuals partially supports hypothesis 4, in line with traits pointing toward lower Conscientiousness for the over-confident (Paulhus and Williams, 2002; Campbell et al., 2004; Jain and Bearden, 2011; O’Boyle et al., 2015). This suggests that both under- and over-confident individuals are less likely to achieve in educational settings. Ac provides an assessment of an individual’s achievement potential and desire to perform in structured situations and predicts mastery of school and higher educational material (Gough, 1964; Repapi et al., 1983; Gough and Lanning, 2016). The Ac construct also has relevance to workplaces, such that executives who score highly on this scale are more hard-working and reliable, but demonstrate tendencies toward risk-aversion, while those who are low on Ac might be viewed as more careless, and rank higher on measures reflecting Neuroticism (Gough and Bradley, 2005). This suggests a potential explanation for our findings with Ac, such that over-confident individuals have lower Ac scores due to a shared association with lower Conscientiousness. In contrast, under-confident individuals show decreased Ac because of their increased Neuroticism. In contrast to Ac, Ai measures the potential for originality and independent thinking in ambiguous situations. Such independence was demonstrated in subsequent behaviors by Gough and Bradley (2005), with those lower in Ai preferring the guidance of additional training that structured learning environments at both tertiary education and workplace training can provide. Low scores on the Ai scale reflect unambitious, simple individuals with a more narrow set of interests (Gough and Bradley, 2005). This description appears to apply to both under- and over-confident individuals in this sample. These findings suggest that under- and over-confident individuals alike are low on both Ac and Ai, pointing to potential difficulties supporting such employees to improve their performance on work tasks.

Further, we might expect both over- and under-confident employees to engage in behavior that isolates them from their fellow employees, as reflected in their lower Social Presence (Sp) and Communality (Cm) scores, a finding that is particularly relevant in the face of working environments that require more employee interaction. The Social Presence (Sp) scale reflects both self-assurance and a need for attention in high scorers, while low scorers on this trait are socially inhibited, unsure of themselves, and concerned about making mistakes (Gough and Bradley, 2005). Social Presence (Sp) was implicated in hypothesis 1, suggesting that over-confident individuals should have higher scores on *Dealing with Others* scales of the CPI260. While the model for Social Presence (Sp) was the only one of these scales to show evidence of significant polynomial effects, the results indicated that over-confident and under-confident individuals alike had *lower* Social Presence (Sp) scores. This suggests that both under- and over-confident individuals were less comfortable being the center of attention, were less self-assured and had fewer social skills. This was unexpected given the suggestion from previous literature that over-confident individuals should be higher in personality characteristics reflecting Extraversion (Schaefer et al., 2004), a trait that is supposed to translate to comfort and enjoyment in social situations. From our results, it appears that people with discrepancies between accuracy and confidence in either direction will be less comfortable being the center of attention, less self-assured, and feel like they have fewer social skills.

Both over- and under-confident individuals also rated themselves as lower on Communality (Cm), a trait describing an individual who expresses conventional, socially accepted thinking, in contrast to low scorers who might be more disorderly, absent-minded, and potentially careless. This finding directly contradicted our expectation that, in line with positive associations between Agreeableness and Communality (Cm), we might expect to see increased scores on this scale as the discrepancy between accuracy and confidence increased in both directions (Sukenik et al., 2018; Mayer et al., 2020). Scores between 30 and 50 *T*-score points indicate unconventional attitudes and inner conflict, such that people scoring low on this scale might be described as distractible, absent-minded, and disorderly (Gough and Bradley, 2005). Those with under-confidence had a *T*-score of 33.27, as compared with over-confident individuals with a *T*-score of 45.88. Although both discrepancies resulted in lower scores on Communality, under-confident individuals were more than one standard deviation below the mean, and more than one standard deviation below those who were over-confident. This suggests a picture of the under-confident individual as more distractable, disorderly, and less able to connect with other employees due to their less conventional thinking styles. However, both over- and under-confident individuals were lower in Communality than the rest of the sample, suggesting a general picture of these individuals as unconventional, irresponsible, and potentially careless. We further did not identify any significant effects along the line of agreement (surface tests *a*_1_ and *a*_2_, see Table 2), indicating no support for hypotheses 5 and 6 that the self-insight of more accurate individuals would be associated with indices of Openness or Conscientiousness, respectively (see surface test *a*_1_ in Table 2).

This research has potential implications for workplace practitioners. Knowing more about the enduring patterns of thought, feeling, and behavior encompassed by certain decision-making styles might allow organizations to ameliorate a problematic decision-making style by providing suitable decision-making supports (Rebele et al., 2021). Both over-confident and under-confident individuals believe they have issues with social situations. However, over-confidence is generally an advantage in social situations, such that individuals will often prefer the advice of an over-confident to a hesitant decision-maker. This was evident in the work of Price and Stone (2004), who found that people preferred financial advisors who expressed more confidence in their investment advice, regardless of the utility of hesitation in such situations. Individuals exhibiting over-confidence might benefit from being coached on this social influencing advantage so that they are more aware of it. In contrast, a lack of social confidence might be expected for under-confident individuals, who we expected to exhibit introverted tendencies. Giving under-confident individuals more independent work tasks (Grant et al., 2011) and supporting them to contribute to group tasks (Barry and Stewart, 1997; Grant et al., 2011), might be helpful for these individuals. Further, there is some evidence to suggest that under-confident individuals respond negatively to socially competitive environments (Canning et al., 2019). Addressing competition between employees might also assist under-confident individuals to succeed.

Thirdly, potential strategies for dealing with confidence bias could be tested in organizational settings based on the current study. Generally, over-confident individuals believe they have performance advantages where in fact they do not. However, this over-confidence effect is directly contradicted by the findings in this study, where over-confident individuals have indicated they believe that they have lower potential to achieve. In contrast to research suggesting that over-confident individuals are unaware of their riskier decision-making style (Jackson and Kleitman, 2014; Jackson et al., 2016, 2017), this study suggests that they might be aware of their achievement derailers. Notably, both under-confident and over-confident individuals rated themselves as lower on achievement potential in both structured and ambiguous situations. Given that under-confident individuals generally have higher accuracy on cognitive tests, we might expect these employees to perform very well but be unaware of this performance advantage. Such employees might benefit from regular feedback from multiple sources to make them aware of their performance advantages (Stankov and Crawford, 1997; Chapman, 2015). In contrast, those who are over-confident might be expressing a justified opinion that they have lower capacity to achieve, based on their lower scores on cognitive tests. Such employees might be supported through performance management processes that help them to overcome their perceived deficiencies in achievement. However, it is equally possible that both under-confident and over-confident individuals are not performing as well as other employees. Such a possibility should be verified in future research using supervisor performance ratings or other objective indicators of work performance.

One trend apparent in these results with relevance to organizational development is the lower self-reported ratings on all four CPI260 scales, for under-confident as compared to over-confident participants. These findings suggest that the under-confident may be particularly prone to experiencing more inner conflict, and may be viewed by others as more anxious, dissatisfied, and pessimistic compared to others. They are unlikely to make a favorable impression on their fellow co-workers due to their lower Social Presence (Sp) scores. Consequently, under-confident employees might have difficulty working on ambiguous tasks where groupwork is required, which is not compensated for by the social advantages conveyed to over-confident employees. These findings suggest they are less likely to demonstrate action-orientated behaviors or display an assertive, expressive interpersonal style that could help them to achieve good outcomes in interpersonal work tasks. The above combination of behaviors is aligned with those typically associated with the Imposter Phenomenon (IP; Clance and Imes, 1978; Bravata et al., 2019). An individual with IP has feelings of fraudulence and inadequacy despite evidence of their achievements. While they possess the intellectual capacity to contribute effectively at senior levels, they may not reach their potential and may not emerge as leaders (Vergauwe et al., 2014). Future research might wish to consider this possibility, using an accuracy and confidence measure together with a validated imposter phenomenon scale (Mak et al., 2019) to establish whether under-confidence shares dispositional variation with the tendency to experience imposter feelings.

Future research examining relative matches and mismatches between ability and confidence and its consequences for personality traits should further consider using polynomial regression with response surface analysis (Shanock et al., 2010; Nestler et al., 2015; Barranti et al., 2017). As far as we are aware, this study is one of the first to apply polynomial regression to the analysis of data including measures of ability and confidence. This is a major methodological strength of the current manuscript, particularly considering the non-linear effects we identified along the line of disagreement (surface test *a*_4_). Previous research using polynomial regression with response surface analysis has examined the role of self-insight into emotional and cognitive abilities in predicting indicators of good adjustment (He and Côté, 2019; Humberg et al., 2019). However, both studies used self-estimates of ability, rather than confidence ratings after each ability question was administered. By examining the combination of ability and confidence using response surface analysis, we were able to preserve the information in the original accuracy and confidence variables. In contrast to the use of difference scores traditional in previous literature (Stone et al., 2001; Campbell et al., 2004; Schaefer et al., 2004; Jain and Bearden, 2011; Sukenik et al., 2018; Mayer et al., 2020) and the use of moderated regression sometimes suggested as an alternative (Cunningham et al., 2018), we were able to explicitly test how all combinations of ability and confidence were associated with personality traits.

## 5. Conclusion

We sought to examine the possibility that confidence bias was meaningfully associated with personality traits using an organizationally relevant personality measure, the CPI260 (Gough and Bradley, 2005). In a high-ability sample of working adults, we identified both under- and over-confidence in measured cognitive abilities. Discrepancies between accuracy and confidence in either direction predicted some personality traits from the California Psychological Inventory, such that working adults who were both over- and under-confident in their measured abilities had less achievement potential in both structured and ambiguous situations, were more socially inhibited, and expressed more unconventional views that likely further isolated them from their working peers. However, this study was also subject to some limitations that were primarily associated with retrospectively analyzing data provided to us from organizational databases. The database available for this study was collected from participants across two national cohorts in Australia and the USA, with limited demographic information to contextualize the findings, and a relatively small sample size for testing polynomial effects. A further limitation with this study was that all individuals in this sample were high achievers, as indicated by the high standardized mental ability score of 111. Standardized cognitive ability scores usually have a mean score of 100 and a standard deviation of fifteen. While the range of scores included participants from 81 to 131, a majority of the sample were located above the normative mean score of 100. This is a potential explanation for why over-confident individuals had the same pattern of personality traits as under-confident individuals. Future research should verify our findings using a proactively designed study, including a larger sample size across a range of cognitive abilities embedded in a single culture, with more targeted questions on demographics.

## Data availability statement

The data analyzed in this study is subject to the following licenses/restrictions: The datasets for this study were provided by Lewis Cadman Consulting and Consulting Psychologists Press. They are not available for analysis by other researchers. Requests to access these datasets should be directed to HD, heather.douglas@newcastle.edu.au.

## Ethics statement

The studies involving human participants were reviewed and approved by the University of Newcastle Human Research Ethics Committee. Written informed consent for participation was not required for this study in accordance with the national legislation and the institutional requirements.

## Author contributions

HD conceptualized the study and drafted the manuscript for publication. MC and JT analyzed the data and contributed to drafts of the method and results. JA reviewed data analysis, provided expert assistance interpreting the CPI260 results, and contributed to drafting the manuscript. All authors contributed to the article and approved the submitted version.
